# Supplementary material for: Three-dimensional synaptic organization of the human hippocampal CA1 field
Source: eLife. 2020 Jul 21;9:e57013. doi: 10.7554/eLife.57013 (PMC7375818; doi:10.7554/eLife.57013)
Supplement: Supplementary file 1. [file elife-57013-supp1.pdf]

	Thickness (mm; mean±SD)	Percentage of all CA1 layers	V_{bv} (%; mean±SD)	V_g (%; mean±SD)	V_{neu} (%; mean±SD)	V_n (%; mean±SD)
SO	0.06±0.03	2%	7.58±3.29	1.95±0.83	0.46±0.47	90.01±3.07
SP	1.13±0.33	42%	5.06±0.99	0.60±0.30	4.23±1.07	90.11±1.32
SR	0.55±0.31	20%	4.79±0.75	0.88±0.75	0.15±0.20	94.19±1.17
SLM	0.62±0.16	23%	5.96±1.25	1.04±0.78	0.04±0.03	92.95±0.73
All layers	2.70±0.62	100%	-	-	-	-

Supplementary file 1A.

Data on CA1 thickness and volume fraction occupied by different cortical elements per layer. SD: standard deviation; SLM: *stratum lacunosum-moleculare*; SO: *stratum oriens*; SP: *stratum pyramidale*; SR: *stratum radiatum*; V_{bv}: volume fraction occupied by blood vessels; V_g: volume fraction occupied by glia; V_{neu}: volume fraction occupied by neurons; V_n: volume fraction occupied by neuropil.

	SO	dSP	sSP	SR	SLM	Totals
Axospinous AS	**1,359 (76.48%)**	**1,788 (81.16%)**	**2,184 (87.61%)**	**1,278 (78.36%)**	**760 (56.80%)**	**7,369 (78.04%)**
On the head	1,354	1,784	2,178	1,273	759	7,348
On the neck	5	4	6	5	1	21
Axospinous SS	**9 (0.51%)**	**37 (1.68%)**	**17 (0.68%)**	**6 (0.37%)**	**31 (2.32%)**	**100 (1.06%)**
On the head	7	30	11	5	29	82
On the neck	2	7	6	1	2	18
Axodendritic AS	**259 (14.58%)**	**202 (9.17%)**	**131 (5.25%)**	**188 (11.53%)**	**300 (22.42%)**	**1,080 (11.44%)**
On spiny shafts	165	107	82	95	81	530
On aspiny shafts	94	95	49	93	219	550
Axodendritic SS	**150 (8.44%)**	**176 (7.99%)**	**161 (6.46%)**	**159 (9.75%)**	**247 (18.46%)**	**893 (9.46%)**
On spiny shafts	98	144	121	114	104	581
On aspiny shafts	52	32	40	45	143	312
Axospinous AS+SS	**1,368 (76.98%)**	**1,825 (82.84%)**	**2,201 (88.29%)**	**1,284 (78.72%)**	**791 (59.12%)**	**7,469 (79.10%)**
On the head	1,361 (76.59%)	1,814 (82.34%)	2,189 (87.81%)	1,278 (78.36%)	788 (58.89%)	7,430 (78.69%)
On the neck	7 (0.39%)	11 (0.50%)	12 (0.48%)	6 (0.37%)	3 (0.22%)	39 (0.41%)
Axodendritic AS+SS	**409 (23.02%)**	**378 (17.16%)**	**292 (11.71%)**	**347 (21.28%)**	**547 (40.88%)**	**1,973 (20.90%)**
On spiny shafts	263 (14.80%)	251 (11.39%)	203 (8.14%)	209 (12.81%)	185 (13.83%)	1,111 (11.77%)
On aspiny shafts	146 (8.22%)	127 (5.76%)	89 (3.57%)	138 (8.46%)	362 (27.06%)	862 (9.13%)
Total AS+SS	**1,777 (100%)**	**2,203 (100%)**	**2,493 (100%)**	**1,631 (100%)**	**1,338 (100%)**	**9,442 (100%)**

Supplementary file 1B.

Postsynaptic target information in all layers of CA1. More detailed information on axospinous synapses can be found in Supplementary file 1I. AS: asymmetric synapses; dSP: deep part of *stratum pyramidale*; SLM: *stratum lacunosum-moleculare*; SO: *stratum oriens*; SR: *stratum radiatum*; SS: symmetric synapses; sSP: superficial part of *stratum pyramidale.*

	Type of postsynaptic target				
	Axospinous AS	Axodendritic AS	Axodendritic SS	Axospinous SS	Totals
SO	**1,359** (1,386.86)	**259** (203.26)	**150** (168.06)	**9** (18.82)	1,777
dSP	**1,788** (1,719.33)	**202** (251.98)	**176** (208.35)	**37** (23.33)	2,203
sSP	**2,184** (1,945.66)	**131** (285.16)	**161** (235.78)	**17** (26.40)	2,493
SR	**1,278** (1,272.91)	**188** (186.56)	**159** (154.26)	**6** (17.27)	1,631
SLM	**760** (1,044.24)	**300** (153.04)	**247** (126.54)	**31** (14.17)	1,338
Totals	7,369	1,080	893	100	9,442

Supplementary file 1C.

A 5x4 contingency table generated to compare the proportion of synapses according to their postsynaptic targets between all layers of CA1. The observed counts of synapses in each subcategory are shown in bold. The expected counts (in parentheses) are calculated from the marginal totals, assuming the null hypothesis that there is no association between the layer and the type of postsynaptic target. A χ^2 test of association was applied to the table, indicating that the null hypothesis must be rejected (p<0.0001). Further 2x2 contingency tables (40 in total; some examples are shown in Supplementary files 1D–1G) were created in order to find the exact layers and postsynaptic targets presenting differences in prevalence, comparing each layer to one another and to every type of postsynaptic target. Similar tables were created to compare all CA1 layers in the rest of the categories analyzed (e.g., excitatory vs inhibitory contacts, synapse subtypes based on the shape of their synaptic junction, etc.). AS: asymmetric synapses; dSP: deep part of *stratum pyramidale*; SLM: *stratum lacunosum-moleculare*; SO: *stratum oriens*; SR: *stratum radiatum*; SS: symmetric synapses; sSP: superficial part of *stratum pyramidale.*

	Type of postsynaptic target		
	Axospinous AS	Other synaptic types	Totals
SO	**1,359** (1,405.08)	**418** (371.92)	1,777
dSP	**1,788** (1,741.92)	**415** (461.08)	2,203
Totals	3,147	833	3,980

Supplementary file 1D.

2x2 contingency table generated after the 5x4 contingency table analysis to compare the proportion of axospinous AS between layers SO and dSP. A χ^2 test of association was applied to the table, indicating that the null hypothesis must be rejected ($p<0.001$).

	Type of postsynaptic target		
	Axodendritic AS	Other synaptic types	Totals
SO	**259** (205.83)	**1,518** (1,571.17)	1,777
dSP	**202** (255.17)	**2,001** (1,947.83)	2,203
Totals	461	3,519	3,980

Supplementary file 1E.

2x2 contingency table generated after the 5x4 contingency table analysis to compare the proportion of axodendritic AS between layers SO and dSP. A χ^2 test of association was applied to the table, indicating that the null hypothesis must be rejected ($p<0.0001$).

	Type of postsynaptic target		
	Axodendritic SS	Other synaptic types	Totals
SO	**150** (145.55)	**1,627** (1,631.45)	1,618
dSP	**176** (180.45)	**2,027** (2,022.55)	1,466
Totals	326	3,654	3,980

Supplementary file 1F.

2x2 contingency table generated after the 5x4 contingency table analysis to compare the proportion of axodendritic SS between layers SO and dSP. A χ^2 test of association was applied to the table, indicating that the null hypothesis must be accepted ($p>0.05$).

	Type of postsynaptic target		
	Axospinous SS	Other synaptic types	Totals
SO	**9**<br>(19.05)	**1,768**<br>(1,755.95)	1,775
dSP	**37**<br>(24.95)	**2,287**<br>(2,299.05)	2,324
Totals	44	4,055	4,099

Supplementary file 1G.

2x2 contingency table generated after the 5x4 contingency table analysis to compare the proportion of axospinous SS between layers SO and dSP. A χ^2 test of association was applied to the table, indicating that the null hypothesis must be rejected ($p<0.001$).

		SO	dSP	sSP	SR	SLM	Totals
AS population	Axospinous AS	1,359 (83.99%)	1,788 (89.85%)	2,184 (94.34%)	1,278 (87.18%)	760 (71.70%)	7,369 (87.22%)
	Axodendritic AS	259 (16.01%)	202 (10.15%)	131 (5.66%)	188 (12.82%)	300 (28.30%)	1,080 (12.78%)
	Total	1,618 (100%)	1,990 (100%)	2,315 (100%)	1,466 (100%)	1,060 (100%)	8,449 (100%)
SS population	Axospinous SS	9 (5.66%)	37 (17.37%)	17 (9.55%)	6 (3.64%)	31 (11.15%)	100 (10.07%)
	Axodendritic SS	150 (94.34%)	176 (82.63%)	161 (90.45%)	159 (96.36%)	247 (88.85%)	893 (89.93%)
	Total	159 (100%)	213 (100%)	178 (100%)	165 (100%)	278 (100%)	993 (100%)
Axospinous synapses	AS	1,359 (99.34%)	1,788 (97.97%)	2,184 (99.23%)	1,278 (99.53%)	760 (96.08%)	7,369 (98.66%)
	SS	9 (0.66%)	37 (2.03%)	17 (0.77%)	6 (0.47%)	31 (3.92%)	100 (1.34%)
	Total	1,368 (100%)	1,825 (100%)	2,201 (100%)	1,284 (100%)	791 (100%)	7,469 (100%)
Axodendritic synapses	AS	259 (63.33%)	202 (53.44%)	131 (44.86%)	188 (54.18%)	300 (54.84%)	1,080 (54.74%)
	SS	150 (36.67%)	176 (46.56%)	161 (55.14%)	159 (45.82%)	247 (45.16%)	893 (45.26%)
	Total	409 (100%)	378 (100%)	292 (100%)	347 (100%)	547 (100%)	1,973 (100%)

Supplementary file 1H.

Postsynaptic target information of AS and SS in all layers of CA1. More detailed information on axospinous synapses can be found in Supplementary file 1I. AS: asymmetric synapses; dSP: deep part of *stratum pyramidale*; SLM: *stratum lacunosum-moleculare*; SO: *stratum oriens*; SR: *stratum radiatum*; SS: symmetric synapses; sSP: superficial part of *stratum pyramidale.*

	SO	dSP	sSP	SR	SLM	CA1
Spines with one synapse	1,338 (98.89%)	1,740 (97.64%)	2,118 (98.10%)	1,240 (98.26%)	717 (95.09%)	7,153 (97.89%)
1 AS	1,338	1,733	2,118	1,240	713	7,142
1 SS	0	7	0	0	4	11
Spines with multiple synapses	15 (1.11%)	42 (2.36%)	41 (1.90%)	22 (1.74%)	37 (4.91%)	157 (2.11%)
2 AS	6	11	23	16	10	66
1 AS+1 SS	9	30	17	6	27	89
3 AS	0	1	1	0	0	2
Spines with 1 head	1,327 (99.03%)	1,743 (98.03%)	2,117 (98.37%)	1,246 (98.75%)	752 (99.75%)	7,185 (98.61%)
Spines with multiple heads	13 (0.97%)	35 (1.97%)	35 (1.63%)	16 (1.25%)	2 (0.25%)	101 (1.39%)
2 Heads	12	34	33	16	2	97
3 Heads	1	1	2	0	0	4

Supplementary file 1I.

Data on dendritic spines in each layer of CA1. AS: asymmetric synapses; CA: *cornu ammonis*; dSP: deep part of *stratum pyramidale*; SLM: *stratum lacunosum-moleculare*; SO: *stratum oriens*; SR: *stratum radiatum*; SS: symmetric synapses; sSP: superficial part of *stratum pyramidale.*

	Type of synapse	No. macular synapses	No. horseshoe-shaped synapses	No. perforated synapses	No. fragmented synapses
SO	AS	2,280 (86.10%)	86 (3.25%)	250 (9.44%)	32 (1.21%)
	SS	133 (80.12%)	22 (13.25%)	8 (4.82%)	3 (1.81%)
dSP	AS	3,294 (85.58%)	141 (3.66%)	357 (9.28%)	57 (1.48%)
	SS	215 (76.51%)	39 (13.88%)	24 (8.54%)	3 (1.07%)
sSP	AS	4,517 (87.15%)	212 (4.09%)	371 (7.16%)	83 (1.60%)
	SS	164 (83.67%)	16 (8.16%)	6 (3.06%)	10 (5.10%)
SR	AS	3,338 (87.02%)	150 (3.91%)	274 (7.14%)	74 (1.93%)
	SS	141 (81.98%)	21 (12.21%)	3 (1.74%)	7 (4.07%)
SLM	AS	2,160 (82.38%)	189 (7.21%)	222 (8.47%)	51 (1.95%)
	SS	258 (81.65%)	37 (11.71%)	13 (4.11%)	8 (2.53%)
Totals	AS	15,589 (85.95%)	778 (4.29%)	1,474 (8.13%)	297 (1.64%)
	SS	911 (80.55%)	135 (11.94%)	54 (4.77%)	31 (2.74%)

Supplementary file 1J.

Proportion of AS and SS according to the shape of their synaptic junction in every layer of CA1. AS: asymmetric synapses; dSP: deep part of *stratum pyramidale*; SLM: *stratum lacunosum-moleculare*; SO: *stratum oriens*; SR: *stratum radiatum*; SS: symmetric synapses; sSP: superficial part of *stratum pyramidale.*

	Type of synapse	No. macular synapses	No. horseshoe-shaped synapses	No. perforated synapses	No. fragmented synapses
SO	Axospinous AS	1,134 (83.44%)	51 (3.75%)	156 (11.48%)	18 (1.32%)
	Axodendritic AS	231 (89.19%)	9 (3.47%)	17 (6.56%)	2 (0.77%)
dSP	Axospinous AS	1,480 (82.77%)	74 (4.14%)	211 (11.80%)	23 (1.29%)
	Axodendritic AS	177 (87.62%)	6 (2.97%)	19 (9.41%)	0 (0.00%)
sSP	Axospinous AS	1,822 (83.42%)	113 (5.17%)	213 (9.75%)	36 (1.65%)
	Axodendritic AS	115 (87.79%)	4 (3.05%)	10 (7.63%)	2 (1.53%)
SR	Axospinous AS	1,042 (81.53%)	61 (4.77%)	145 (11.35%)	30 (2.35%)
	Axodendritic AS	170 (90.43%)	7 (3.72%)	7 (3.72%)	4 (2.13%)
SLM	Axospinous AS	610 (80.26%)	68 (8.95%)	67 (8.82%)	15 (1.97%)
	Axodendritic AS	202 (67.33%)	27 (9.00%)	67 (22.33%)	4 (1.33%)
Totals	Axospinous AS	6,088 (82.62%)	367 (4.98%)	792 (10.75%)	122 (1.66%)
	Axodendritic AS	895 (82.87%)	53 (4.91%)	120 (11.11%)	12 (1.11%)

Supplementary file 1K.

Proportion of AS according to the shape of their synaptic junction within the axospinous and axodendritic synaptic population in every layer of CA1. AS: asymmetric synapses; dSP: deep part of *stratum pyramidale*; SLM: *stratum lacunosum-moleculare*; SO: *stratum oriens*; SR: *stratum radiatum*; SS: symmetric synapses; sSP: superficial part of *stratum pyramidale.*

	Area of SAS AS (nm^2; mean±sem)	Perimeter of SAS AS (nm; mean±sem)	Curvature of SAS AS (mean±sem)	Area of SAS SS (nm^2; mean±sem)	Perimeter of SAS SS (nm; mean±sem)	Curvature of SAS SS (mean±sem)
SO	86,716.52±1,371.02	1,416.85±14.82	0.045±0.001	85,737.60±5,869.60	1,561.04±65.55	0.044±0.002
dSP	92,045.29±1,192.92	1,467.14±12.26	0.048±0.001	74,764.69±3,057.33	1,494.82±45.84	0.044±0.001
sSP	88,061.63±1,038.49	1,456.89±10.76	0.053±0.001	58,305.43±2,612.01	1,257.36±37.28	0.055±0.002
SR	82,841.26±1,201.47	1,389.57±12.63	0.052±0.001	63,183.20±2,734.96	1,344.78±42.85	0.050±0.002
SLM	91,419.95±1,376.38	1,477.28±14.28	0.044±0.001	57,390.19±2,071.04	1,288.54±33.29	0.046±0.001
Totals (CA1)	89,727.65±5,775.90	1,458.82±56.17	0.050±0.002	67,236.17±4,456.52	1,378.38±71.47	0.047±0.002

	AS					SS				
	Log-logistic 3P			Log-normal		Log-logistic 3P			Log-normal	
	α	β	γ	σ	μ	α	β	γ	σ	μ
SO	2.36	61,628	4,445.8	0.69	11.12	2.48	65,225	1,070.3	0.71	11.10
dSP	2.36	66,569	3,670.4	0.71	11.18	2.59	62,274	-919.4	0.68	11.00
sSP	2.36	63,446	2,550.3	0.72	11.12	1.86	40,694	2,684.8	0.85	10.67
SR	2.26	57,818	2,874.7	0.74	11.04	2.24	51,574	-1,709.9	0.80	10.77
SLM	2.32	65,617	4,608.1	0.69	11.18	2.90	50,419	-624.3	0.61	10.80
Totals (CA1)	2.35	63,425	2,960.2	0.72	11.12	2.48	55,292	-1,350.5	0.74	10.87

Supplementary file 1L.

Morphological data on synapses based on synaptic apposition surface (SAS) in all layers of CA1.

In the bottom table, the shape (α), scale (β) and location (γ) of the best-fit log-logistic 3P distribution and the scale (σ) and location (μ) of the best-fit log-normal distribution of the SAS area in each layer and the whole CA1 are indicated. AS: asymmetric synapses; CA: *cornu ammonis*; dSP: deep part of *stratum pyramidale*; sem: standard error of the mean; SLM: *stratum lacunosum-*

moleculare; SO: *stratum oriens*; SR: *stratum radiatum*; SS: symmetric synapses; sSP: superficial part of *stratum pyramidale.*

		Area of SAS AS (nm^2; mean±sem)	Perimeter of SAS AS (nm; mean±sem)	Curvature of SAS AS (mean±sem)	Area of SAS SS (nm^2; mean±sem)	Perimeter of SAS SS (nm; mean±sem)	Curvature of SAS SS (mean±sem)
SO	Axospinous	92,900.02±4,207.11	1,490.11±42.11	0.045±0.001	76,841.78±24,870.78	1,428.24±244.41	0.042±0.003
	Axodendritic	111,843.21±15,119.25	1,626.09±127.58	0.045±0.004	88,155.34±13,916.54	1,579.45±122.73	0.044±0.002
dSP	Axospinous	100,377.53±3,285.52	1,554.92±39.94	0.049±0.003	53,628.30±8,432.84	1,170.13±99.05	0.039±0.003
	Axodendritic	113,415.07±11,395.55	1,681.01±121.94	0.039±0.003	82,319.21±4,124.52	1,629.20±111.60	0.044±0.002
sSP	Axospinous	101,533.55±10,258.19	1,588.45±93.27	0.053±0.001	45,484.55±3,458.52	995.51±56.06	0.048±0.007
	Axodendritic	83,216.23±11,310.38	1,392.52±111.42	0.049±0.005	64,386.67±9,153.64	1,327.90±108.50	0.053±0.003
SR	Axospinous	102,259.93±11,303.07	1,586.06±101.93	0.054±0.001	52,408.99±8,678.45	1,173.23±24.75	0.078±0.027
	Axodendritic	99,896.11±23,037.14	1,514.68±205.07	0.047±0.003	60,472.43±7,514.73	1,291.74±115.58	0.050±0.005
SLM	Axospinous	98,081.18±6,717.02	1,567.25±68.94	0.043±0.002	41,129.57±4,778.92	1,063.43±111.81	0.055±0.009
	Axodendritic	159,586.41±10,174.50	2,041.64±77.33	0.046±0.002	62,468.75±3,482.63	1,371.04±78.01	0.044±0.002
Totals	Axospinous	98,200.61±6,202.57	1,548.38±63.25	0.050±0.001	49,044.59±5,442.64	1,126.06±87.34	0.048±0.003
	Axodendritic	117,360.02±8,315.30	1,686.99±82.06	0.045±0.002	71,218.23±4,426.35	1,436.55±68.97	0.047±0.002

Supplementary file 1M.

Morphological data based on synaptic apposition surface (SAS) regarding the postsynaptic targets in every layer of CA1. AS: asymmetric synapses; dSP: deep part of *stratum pyramidale*; sem: standard error of the mean; SLM: *stratum lacunosum-moleculare*; SO: *stratum oriens*; SR: *stratum radiatum*; SS: symmetric synapses; sSP: superficial part of *stratum pyramidale*.

		Area of AS SAS AS (nm^2; mean±sem)	Perimeter of SAS AS (nm; mean±sem)	Curvature of SAS AS (mean±sem)	Area of SAS SS (nm^2; mean±sem)	Perimeter of SAS SS (nm; mean±sem)	Curvature of SAS SS (mean±sem)
SO	Macular	74,181.28±4,910.43	1,283.10±58.28	0.043±0.002	72,168.70±10,458.36	1,330.32±104.11	0.043±0.002
	Horseshoe-shaped	171,171.18±8,988.19	2,787.25±140.87	0.065±0.006	101,225.81±10,559.25	2,078.29±213.47	0.041±0.004
	Perforated	188,978.45±13,785.62	2,426.28±124.62	0.055±0.008	233,367.89±77,405.62	3,306.30±766.80	0.064±0.022
	Fragmented	223,086.00±10,491.93	2,542.75±180.04	0.099±0.015	167,220.54±41,188.75	2,187.00±204.15	0.064±0.006
dSP	Macular	73,347.29±1,957.92	1,272.02±18.33	0.044±0.002	58,981.36±3,424.93	1,244.50±56.04	0.042±0.002
	Horseshoe-shaped	185,893.89±12,000.00	3,046.28±122.58	0.086±0.008	114,528.91±12,807.42	2,300.59±160.87	0.044±0.005
	Perforated	205,732.10±9,992.43	2,560.79±90.50	0.062±0.003	114,415.03±16,821.37	2,071.89±204.10	0.047±0.005
	Fragmented	274,778.26±18,518.33	2,368.79±142.38	0.120±0.013	76,662.93±11,428.32	1,277.38±191.22	0.065±0.022
sSP	Macular	70,946.54±5,240.96	1,276.40±50.50	0.048±0.001	51,365.93±5,150.52	1,132.33±53.80	0.053±0.002
	Horseshoe-shaped	213,184.36±24,896.05	3,229.59±262.31	0.092±0.008	99,774.37±13,522.27	2,135.95±172.18	0.046±0.011
	Perforated	225,076.44±26,339.64	2,759.96±232.75	0.078±0.003	118,372.40±38,494.85	2,139.19±366.81	0.031±0.005
	Fragmented	266,724.88±29,303.55	2,563.51±172.67	0.131±0.017	110,887.92±32,290.73	1,600.21±309.21	0.071±0.003
SR	Macular	65,852.36±3,067.58	1,205.12±25.97	0.048±0.004	51,088.37±4,910.94	1,128.12±54.26	0.051±0.006
	Horseshoe-shaped	181,125.70±17,161.52	2,854.53±230.13	0.071±0.003	105,630.16±11,089.39	2,147.72±257.86	0.034±0.010
	Perforated	201,824.60±23,993.63	2,638.11±248.50	0.076±0.004	63,548.92±27,451.37	1,328.90±466.08	0.044±0.013
	Fragmented	269,475.34±35,799.23	2,683.22±317.04	0.118±0.014	89,588.42±11,515.62	1,563.85±63.03	0.049±0.003
SLM	Macular	72,110.84±2,661.42	1,247.41±16.51	0.041±0.002	50,704.33±10,595.42	1,184.14±33.54	0.047±0.003
	Horseshoe-shaped	160,347.91±12,123.34	2,673.00±86.45	0.054±0.003	65,798.60±10,255.21	1,630.59±168.00	0.041±0.003
	Perforated	201,177.54±15,526.48	2,542.94±79.43	0.057±0.002	95,746.01±14,562.01	1,933.97±218.85	0.036±0.004

	Fragmented	202,913.98±8,401.89	2,199.00±87.97	0.077±0.009	149,534.82±11,567.29	1,834.22±244.95	0.062±0.002
Totals	Macular	70,322.92±2,524.70	1,251.59±27.36	0.045±0.002	56,769.81±2,226.39	1,204.01±37.92	0.047±0.003
	Horseshoe-shaped	146,474.80±8,979.65	2,908.08±132.62	0.075±0.003	100,178.06±4,541.53	2,132.38±75.35	0.044±0.003
	Perforated	205,500.73±13,875.57	2,598.53±137.87	0.067±0.001	135,419.88±24,500.18	2,206.83±210.21	0.047±0.005
	Fragmented	249,642.43±17,666.73	2,480.97±157.13	0.091±0.021	109,912.28±12,528.19	1,655.41±92.67	0.062±0.003

Supplementary file 1N.

Morphological data based on synaptic apposition surface (SAS) of synapses regarding the shape of the synaptic junction in every layer of CA1. AS: asymmetric synapses; dSP: deep part of *stratum pyramidale*; SAS: synaptic apposition surface; sem: standard error of the mean; SLM: *stratum lacunosum-moleculare*; SO: *stratum oriens*; SR: *stratum radiatum*; SS: symmetric synapses; sSP: superficial part of *stratum pyramidale.*

		Thickness (mm; mean±SD)	Percentage of all CA1 layers	V_{bv} (%; mean±SD)	V_g (%; mean±SD)	V_{neu} (%; mean±SD)	V_n (%; mean±SD)
AB1	SO	0.07±0.02	2%	13.03±0.59	1.54±0.70	0.14±0.24	85.30±1.26
	SP	1.66±0.03	49%	6.10±0.33	1.03±0.26	3.80±0.35	89.07±0.20
	SR	0.47±0.11	14%	4.57±0.48	2.19±0.90	0.04±0.08	93.20±1.15
	SLM	0.79±0.04	23%	5.38±0.91	2.03±0.51	0.03±0.04	92.57±1.40
	All layers	3.41±0.06	100%	-	-	-	-
AB2	SO	0.03±0.01	1%	8.15±2.99	2.32±0.71	0.00±0.00	89.53±2.52
	SP	1.07±0.03	47%	4.88±0.33	0.60±0.31	4.94±0.39	89.57±0.31
	SR	0.27±0.01	12%	6.04±1.21	0.80±0.52	0.44±0.22	92.73±1.42
	SLM	0.62±0.05	27%	7.10±0.36	0.36±0.31	0.03±0.05	92.51±0.68
	All layers	2.27±0.03	100%	-	-	-	-
AB3	SO	0.04±0.01	2%	5.50±1.35	3.24±1.87	1.16±1.09	90.10±1.85
	SP	0.82±0.11	39%	4.02±0.81	0.73±0.23	5.69±1.50	89.56±2.44
	SR	0.40±0.06	19%	4.20±0.96	0.56±0.50	0.28±0.09	94.97±0.45
	SLM	0.43±0.05	20%	4.46±1.37	1.67±0.40	0.00±0.00	93.87±1.08
	All layers	2.13±0.08	100%	-	-	-	-
AB4	SO	0.10±0.01	4%	6.42±2.74	1.38±0.28	0.69±0.72	91.51±2.99
	SP	0.90±0.19	38%	6.08±0.19	0.26±0.17	3.71±0.58	89.95±0.73
	SR	0.55±0.08	24%	4.89±0.93	0.53±0.46	0.00±0.00	94.58±1.34
	SLM	0.62±0.01	26%	7.40±1.13	0.29±0.06	0.08±0.13	92.23±1.20
	All layers	2.34±0.08	100%	-	-	-	-
M17	SO	0.05±0.00	1%	4.81±0.59	1.27±0.17	0.30±0.07	93.63±0.61
	SP	1.17±0.11	35%	4.21±1.69	0.37±0.17	3.01±0.30	92.41±1.85
	SR	1.07±0.18	32%	4.24±0.48	0.31±0.27	0.00±0.00	95.46±0.68
	SLM	0.67±0.03	20%	5.47±1.71	0.88±0.19	0.05±0.09	93.60±1.83
	All layers	3.35±0.23	100%	-	-	-	-

Supplementary file 1O.

Data on CA1 thickness and volume fraction occupied by different cortical elements per case. CA: *cornu ammonis*; SD: standard deviation; SLM: *stratum lacunosum-moleculare*; SO: *stratum oriens*; SP: *stratum pyramidale*; SR: *stratum radiatum*; V_{bv}: volume fraction occupied by blood vessels; V_g: volume fraction occupied by glia; V_{neu}: volume fraction occupied by neurons; V_n: volume fraction occupied by neuropil.

The following significant differences were observed between cases:

- SO:
  - V_{bv}:
    - AB1-AB3 (ANOVA, p=0.005)
    - AB1-AB4 (ANOVA, p=0.013)
    - AB1-M17 (ANOVA, p=0.003)
  - V_n:
    - AB1-AB4 (ANOVA, p=0.024)
    - AB1-M17 (ANOVA, p=0.004)
- SP:
  - V_{neu}:
    - AB3-M17 (ANOVA, p=0.011)
- SR:
  - V_{neu}:
    - AB1-AB2 (ANOVA, p=0.004)
    - AB2-AB4 (ANOVA, p=0.002)
    - AB2-M17 (ANOVA, p=0.002)
    - AB3-AB4 (ANOVA, p=0.039)
    - AB3-M17 (ANOVA, p=0.039)
  - V_{glia}:
    - AB1-AB2 (ANOVA, p=0.082)
    - AB1-AB3 (ANOVA, p=0.038)
    - AB1-AB4 (ANOVA, p=0.034)
    - AB1-M17 (ANOVA, p=0.015)
- SLM:
  - V_{glia}:
    - AB1-AB2 (ANOVA, p=0.001)
    - AB1-AB4 (ANOVA, p=0.001)
    - AB1-M17 (ANOVA, p=0.019)
    - AB2-AB3 (ANOVA, p=0.006)
    - AB3-AB4 (ANOVA, p=0.004).

STRATUM ORIENS					
	AB1	AB2	AB3	AB4	M17
No. AS	399	961	446	346	496
No. SS	20	48	40	15	43
% AS	95.23%	95.24%	91.77%	95.84%	92.02%
% SS	4.77%	4.76%	8.23%	4.16%	7.98%
CF volume (µm^3)	1,085	1,281	1,126	1,308	1,422
Density AS/µm^3 (mean±SD)	0.37±0.04	0.75±0.16	0.40±0.11	0.27±0.06	0.35±0.05
Density SS/µm^3 (mean±SD)	0.02±0.00	0.04±0.01	0.03±0.02	0.01±0.01	0.03±0.01
Density AS+SS/µm^3 (mean±SD)	0.39± 0.04	0.78± 0.17	0.43± 0.10	0.28± 0.07	0.38± 0.04
SAS Area of AS (nm^2; mean±sem)	93,342.82± 3,268.05	73,718.73± 1,964.31	94,969.69± 3,940.23	107,520.65 ± 4,444.53	84,635.58± 2,937.08
SAS Perimeter of AS (nm; mean±sem)	1,438.33± 36.58	1,267.48± 21.07	1,513.43± 44.28	1,619.57± 42.70	1,460.72± 33.10
SAS Curvature of AS (mean±sem)	0.046± 0.001	0.044± 0.001	0.046± 0.002	0.051± 0.002	0.040± 0.001
SAS Area of SS (nm^2; mean±sem)	70,229.23± 10,794.99	61,411.26± 6,274.37	139,459.24 ± 18,604.16	88,163.18± 18,312.31	68,925.38± 4,248.17
SAS Perimeter of SS (nm; mean±sem)	1,341.18± 142.26	1,332.72± 96.05	2,039.02± 183.62	1,552.94± 235.24	1,471.26± 79.48
SAS Curvature of SS (mean±sem)	0.048± 0.004	0.045± 0.003	0.047± 0.007	0.045± 0.006	0.037± 0.002
Distance to nearest synapse (nm; mean±SD)	798.45± 71.69	647.61± 48.08	732.96± 48.85	802.36± 37.28	732.66± 11.24

Supplementary file 1P.

Ultrastructural analysis of the neuropil of the *stratum oriens* of CA1 per case. AS: asymmetric synapses; CF: counting frame; No.: number; SAS: synaptic apposition surface; SD: standard deviation; sem: standard error of the mean; SS: symmetric synapses.

The following significant differences were observed between cases:

- Synaptic density:
  - AB1-AB2 (ANOVA, p=0.004)
  - AB2-AB3 (ANOVA, p=0.008)
  - AB2-AB4 (ANOVA, p=0.003)
  - AB2-M17 (ANOVA, p=0.001)
- AS SAS Area:
  - AB2-AB4 (ANOVA, p=0.004)
  - AB4-M17 (ANOVA, p=0.031)
- AS SAS Perimeter:
  - AB2-AB4 (ANOVA, p=0.016)
- AS SAS Curvature:
  - AB4-M17 (ANOVA, p=0.009).

STRATUM PYRAMIDALE (DEEP)					
	AB1	AB2	AB3	AB4	M17
No. AS	528	1168	694	708	751
No. SS	45	103	39	46	48
% AS	92.15%	91.90%	94.68%	93.90%	93.99%
% SS	7.85%	8.10%	5.32%	6.10%	6.01%
CF volume (µm^3)	1181	1241	1061	1281	1239
Density of AS/µm^3 (mean±SD)	0.48±0.06	1.05±0.41	0.69±0.01	0.58±0.05	0.65±0.26
Density of SS/µm^3 (mean±SD)	0.04±0.01	0.08±0.03	0.04±0.01	0.04±0.00	0.04±0.02
Density of AS+SS/µm^3 (mean±SD)	0.48± 0.06	1.05± 0.41	0.69± 0.01	0.58± 0.05	0.65± 0.26
SAS Area of AS (nm^2; mean±sem)	92,722.75± 2,681.12	81,061.60± 2024.49	97,161.24± 3,191.30	101,859.21 ± 2,938.24	94,678.63± 2,678.52
SAS Perimeter of AS (nm; mean±sem)	1,453.48± 27.87	1,333.65± 19.93	1,504.59± 29.95	1,605.18± 32.69	1,519.67± 28.75
SAS Curvature of AS (mean±sem)	0.045± 0.001	0.043± 0.001	0.056± 0.002	0.057± 0.001	0.044± 0.001
SAS Area of SS (nm^2; mean±sem)	67,643.28± 7,427.96	72,582.05± 4,969.33	91,389.02± 10,215.35	69,230.11± 5,762.38	77,574.95± 7,439.81
SAS Perimeter of SS (nm; mean±sem)	1,236.47± 94.11	1,433.30± 65.90	1,870.83± 161.56	1,428.71± 86.55	1,619.03± 128.33
SAS Curvature of SS (mean±sem)	0.041± 0.004	0.047± 0.002	0.044± 0.003	0.046± 0.003	0.037± 0.002
Distance to nearest synapse (nm; mean±SD)	722.14± 27.54	586.62± 59.88	646.90± 5.95	704.29± 4.97	689.09± 93.50

Supplementary file 1Q.

Ultrastructural analysis of the neuropil of the deep part of *stratum pyramidale* of CA1 per case. AS: asymmetric synapses; CF: counting frame; SAS: synaptic apposition surface; SD: standard deviation; sem: standard error of the mean; SS: symmetric synapses.

The following significant differences were observed between cases:

- AS SAS Perimeter:
    - AB2-AB4 (ANOVA, p=0.017)
- AS SAS Curvature:
    - AB2-AB4 (ANOVA, p=0.043)
    - AB4-M17 (ANOVA, p=0.043).

STRATUM PYRAMIDALE (SUP)					
	AB1	AB2	AB3	AB4	M17
No. AS	1,063	1,242	1,180	697	1,001
No. SS	31	43	30	49	43
% AS	97.17%	96.65%	97.52%	93.43%	95.88%
% SS	2.83%	3.35%	2.48%	6.57%	4.12%
CF volume (µm^3)	1,008	1,125	1,114	1,054	1,100
Density of AS/µm^3 (mean±SD)	1.06±.220.08	1.11±0.22	1.06±0.14	0.66±0.10	0.90±0.07
Density of SS/µm^3 (mean±SD)	0.03±0.01	0.04±0.01	0.03±0.01	0.05±0.01	0.04±0.01
Density of AS+SS/µm^3 (mean±SD)	1.09± 0.08	1.14± 0.23	1.09± 0.13	0.71± 0.10	0.94± 0.08
SAS Area of AS (nm^2; mean±sem)	70,955.73± 1,513.22	78,494.59 ± 1,949.28	81,458.42± 1,967.25	120,382.77 ± 3,462.29	103,428.73 ± 2,809.43
SAS Perimeter of AS (nm; mean±sem)	1,327.93± 17.92	1,325.21± 19.52	1,405.83± 20.29	1,726.02± 34.42	1,630.59± 29.97
SAS Curvature of AS (mean±sem)	0.054± 0.001	0.054± 0.001	0.053± 0.001	0.058± 0.001	0.050± 0.001
SAS Area of SS (nm^2; mean±sem)	42,279.64± 4,088.94	4,4082.07 ± 4,942.68	73,446.84± 12,905.52	76,579.79± 10,317.67	64,714.08± 7,793.82
SAS Perimeter of SS (nm; mean±sem)	1,072.17± 61.40	1,110.70± 93.53	1,511.62± 182.48	1,387.66± 126.13	1,283.53± 94.11
SAS Curvature of SS (mean±sem)	0.048± 0.004	0.059± 0.005	0.060± 0.005	0.052± 0.003	0.046± 0.004
Distance to nearest synapse (nm; mean±SD)	589.48± 37.31	571.68± 25.00	579.05± 11.49	665.82± 53.95	613.96± 28.69

Supplementary file 1R.
Ultrastructural analysis of the neuropil of the superficial part of *stratum pyramidale* of CA1 per case. AS: asymmetric synapses; CF: counting frame; SAS: synaptic apposition surface; SD: standard deviation; sem: standard error of the mean; SS: symmetric synapses; Sup: superficial.

The following significant differences were observed between cases:

- AS:SS ratio:
  - AB1-AB4 (χ^2, p=0.0002)
  - AB3-AB4 (χ^2, p=1.475x10^{-5})
- AS SAS Area:
  - AB1-AB4 (ANOVA, p=0.0002)
  - AB1-M17 (ANOVA, p=0.006)
  - AB2-AB4 (ANOVA, p=0.0009)
  - AB2-M17 (ANOVA, p=0.036)
  - AB3-AB4 (ANOVA, p=0.002)
- AS SAS Perimeter:
  - AB1-AB4 (ANOVA, p=6.182x10^{-5})
  - AB1-M17 (ANOVA, p=0.0005)
  - AB2-AB4 (ANOVA, p=6.351x10^{-5})
  - AB2-M17 (ANOVA, p=0.0006)
  - AB3-AB4 (ANOVA, p=0.0004)
  - AB3-M17 (ANOVA, p=0.006).

STRATUM RADIATUM					
	AB1	AB2	AB3	AB4	M17
No. AS	647	897	1048	572	672
No. SS	25	24	39	42	42
% AS	96.28%	97.39%	96.41%	93.16%	94.12%
% SS	3.72%	2.61%	3.59%	6.84%	5.88%
CF volume (μm^3)	1,310	1,075	1,176	1,085	1,362
Density of AS/μm^3 (mean±SD)	0.50±0.03	0.83±0.10	0.87±0.17	0.53±0.11	0.49±0.04
Density of SS/μm^3 (mean±SD)	0.02±0.01	0.02±0.02	0.03±0.02	0.04±0.01	0.03±0.01
Density of AS+SS/μm^3 (mean±SD)	0.52± 0.03	0.85± 0.09	0.91± 0.16	0.57± 0.12	0.53± 0.05
SAS Area of AS (nm^2; mean±sem)	76,444.31± 2,197.02	72,546.21± 2,322.56	72,385.57± 1,854.08	105,184.59 ± 3,515.39	100,176.82 ± 1,201.47
SAS Perimeter of AS (nm; mean±sem)	1,338.18± 22.34	1,295.34± 25.94	1,299.66± 20.15	1,581.08± 36.93	1,543.33± 38.14
SAS Curvature of AS (mean±sem)	0.052± 0.001	0.056± 0.001	0.048± 0.001	0.062± 0.002	0.043± 0.001
SAS Area of SS (nm^2; mean±sem)	52,100.88± 8,050.33	41,303.20± 6,506.56	53,992.48± 7,792.31	64,738.80± 6,049.48	86,398.68± 7,593.45
SAS Perimeter of SS (nm; mean±sem)	1,156.15± 115.84	1,010.78± 107.58	1,177.10± 109.14	1,361.28± 116.76	1,677.58± 125.05
SAS Curvature of SS (mean±sem)	0.045± 0.004	0.065± 0.009	0.050± 0.005	0.057± 0.005	0.035± 0.003
Distance to nearest synapse (nm; mean±SD)	694.96± 15.37	600.94± 8.77	587.65± 46.61	724.52± 31.04	701.98± 43.51

Supplementary file 1S.

Ultrastructural analysis of the neuropil of the *stratum radiatum* of CA1 per case. AS: asymmetric synapses; CF: counting frame; SAS: synaptic apposition surface; SD: standard deviation; sem: standard error of the mean; SS: symmetric synapses.

The following significant differences were observed between cases:

- Synaptic density:
  - AB1-AB2 (ANOVA, p=0.017)
  - AB1-AB3 (ANOVA, p=0.007)
  - AB2-AB4 (ANOVA, p=0.021)
  - AB2-M17 (ANOVA, p=0.044)
  - AB3-AB4 (ANOVA, p=0.008)
  - AB3-M17 (ANOVA, p=0.017)
- AS:SS ratio:
  - AB2-AB4 (χ^2, p=9.165x10^{-5})
  - AB2-M17 (χ^2, p=0.0009)
- AS SAS Area:
  - AB1-AB4 (ANOVA, p=0.012)
  - AB1-M17 (ANOVA, p=0.045)
  - AB2-AB4 (ANOVA, p=0.004)
  - AB2-M17 (ANOVA, p=0.015)
  - AB3-AB4 (ANOVA, p=0.004)
  - AB3-M17 (ANOVA, p=0.015)
- AS SAS Perimeter:
  - AB1-AB4 (ANOVA, p=0.039)
  - AB2-AB4 (ANOVA, p=0.014)
  - AB2-M17 (ANOVA, p=0.038)
  - AB3-AB4 (ANOVA, p=0.016)
  - AB3-M17 (ANOVA, p=0.046)
- AS SAS Curvature:
  - AB1-AB4 (ANOVA, p=0.033)
  - AB2-M17 (ANOVA, p=0.008)
  - AB3-AB4 (ANOVA, p=0.006)
  - AB4-M17 (ANOVA, p=0.0005)
- SS SAS Area:
  - AB2-AB4 (ANOVA, p=0.030)
  - AB2-M17 (ANOVA, p=0.010).

STRATUM LACUNOSUM-MOLECULARE					
	AB1	AB2	AB3	AB4	M17
No. AS	455	533	702	429	503
No. SS	43	40	70	70	93
% AS	91.37%	93.02%	90.93%	85.97%	84.40%
% SS	8.63%	6.98%	9.07%	14.03%	15.60%
CF volume (µm^3)	1,141	1,127	1,278	1,035	1,109
Density of AS/µm^3 (mean±SD)	0.40±0.05	0.47±0.04	0.56±0.15	0.39±0.07	0.49±0.04
Density of SS/µm^3 (mean±SD)	0.04±0.00	0.04±0.01	0.05±0.01	0.06±0.01	0.09±0.01
Density of AS+SS/µm^3 (mean±SD)	0.44± 0.05	0.51± 0.04	0.61± 0.15	0.45± 0.07	0.58± 0.04
SAS Area of AS (nm^2; mean±sem)	88,303.06± 3,115.26	98,697.11± 3,522.76	76,680.19± 2,143.59	112,152.43 ± 4,038.28	89,394.31± 1,376.38
SAS Perimeter of AS (nm; mean±sem)	1,149.67± 33.63	1,507.70± 32.98	1,375.86± 23.90	1,604.79± 39.69	1,502.69± 32.95
SAS Curvature of AS (mean±sem)	0.047± 0.002	0.042± 0.001	0.047± 0.001	0.044± 0.002	0.040± 0.001
SAS Area of SS (nm^2; mean±sem)	47,017.73± 4,264.25	72,916.74± 7,087.22	61,925.95± 5,471.59	53,253.73± 2,769.68	59,848.75± 3,890.73
SAS Perimeter of SS (nm; mean±sem)	1,086.19± 63.88	1,589.32± 129.80	1,446.40± 93.66	1,191.99± 43.82	1,287.05± 53.39
SAS Curvature of SS (mean±sem)	0.046± 0.003	0.045± 0.003	0.050± 0.002	0.053± 0.004	0.038± 0.002
Distance to nearest synapse (nm; mean±SD)	696.49± 22.38	715.96± 52.01	651.23± 49.79	695.65± 20.83	688.92± 31.36

Supplementary file 1T.

Ultrastructural analysis of the neuropil of the *stratum lacunosum-moleculare* of CA1 per case. AS: asymmetric synapses; CF: counting frame; SAS: synaptic apposition surface; SD: standard deviation; sem: standard error of the mean; SS: symmetric synapses.

The following significant differences were observed between cases:

- AS:SS ratio:
  - AB1-M17 (χ^2, p=0.0005)
  - AB2-AB4 (χ^2, p=0.0002)
  - AB2-M17 (χ^2, p=3.332x10^{-6})
  - AB3-M17(χ^2, p=0.0003)
- AS SAS Area:
  - AB3-AB4 (ANOVA, p=0.006)
- SS SAS Area:
  - AB1-AB2 (ANOVA, p=0.026)
- SS SAS Perimeter:
  - AB1-AB2 (ANOVA, p=0.002)
  - AB1-AB3 (ANOVA, p=0.015)
  - AB2-AB4 (ANOVA, p=0.009)
  - AB2-M17 (ANOVA, p=0.048).

STRATUM ORIENS						
		No. AS	No. SS	SAS Area of AS (nm^2; mean±sem)	SAS Perimeter of AS (nm; mean±sem)	SAS Curvature of AS (mean±sem)
Axospinous	AB1	254 (77.44%)	1 (0.30%)	97,732.76±4,179.97	1,484.16±46.83	0.045±0.002
	AB2	455 (87.33%)	4 (0.77%)	81,382.46±3,033.28	1,349.53±32.93	0.043±0.001
	AB3	226 (73.14%)	2 (0.65%)	94,628.46±5,446.79	1,535.91±65.99	0.045±0.002
	AB4	184 (83.26%)	2 (0.90%)	104,936.32±5,177.26	1,605.67±56.16	0.048±0.002
	M17	240 (60.30%)	0 (0.00%)	85,820.10±4,478.71	1,475.30±52.95	0.042±0.002
Axodendritic	AB1	56 (17.07%)	17 (5.18%)	98,681.86±7,172.21	1,449.85±66.75	0.040±0.004
	AB2	22 (4.22%)	40 (7.68%)	69,026.21±11,540.48	1,233.45±145.04	0.048±0.006
	AB3	43 (13.92%)	38 (12.30%)	128,191.12±13,286.59	1,806.62±135.43	0.044±0.003
	AB4	22 (9.95%)	13 (5.88%)	159,160.95±32,555.49	1,947.36±191.55	0.059±0.009
	M17	116 (29.15%)	42 (10.55%)	104,155.92±5,740.68	1,693.18±60.24	0.035±0.002

Supplementary file 1U.
Ultrastructural data regarding the postsynaptic target in the *stratum oriens* of CA1 per case. AS: asymmetric synapses; SAS: synaptic apposition surface; sem: standard error of the mean; SS: symmetric synapses.

The following significant differences were observed between cases:

- AB1-AB2 (χ^2, p=6.119x10^{-9})
  - Axospinous AS (χ^2, p=0.0002)
  - Axodendritic AS (χ^2, p=5.906x10^{-10})
- AB1-M17 (χ^2, p=7.802x10^{-6})
  - Axospinous AS (χ^2, p=8.300x10^{-7})
  - Axodendritic AS (χ^2, p=0.0002)

- AB2-AB3 (χ^2, p=4.150x10^{-7})
    - Axospinous AS (χ^2, p=5.445x10^{-7})
    - Axodendritic AS (χ^2, p=1.509x10^{-6})

- AB2-M17 (χ^2, p=1.000x10^{-17})
    - Axospinous AS (χ^2, p=1.000x10^{-17})
    - Axodendritic AS (χ^2, p=1.000x10^{-17})
- AB3-M17 (χ^2, p=1.315x10^{-5})
    - Axospinous AS (χ^2, p=0.0004)
    - Axodendritic AS (χ^2, p=1.274x10^{-6})
- AB4-M17 (χ^2, p=5.130x10^{-9})
    - Axospinous AS (χ^2, p=1.832x10^{-9})
    - Axodendritic AS (χ^2, p=1.155x10^{-8}).

STRATUM PYRAMIDALE (DEEP)						
		No. AS	No. SS	SAS Area of AS (nm^2; mean±sem)	SAS Perimeter of AS (nm; mean±sem)	SAS Curvature of AS (mean±sem)
Axospinous	AB1	417 (86.69%)	14 (2.91%)	95,332.59±2,932.72	1,479.39±30.01	0.045±0.001
	AB2	357 (67.61%)	6 (1.14%)	92,357.50±3,763.36	1,452.66±37.06	0.044±0.001
	AB3	347 (85.68%)	4 (0.99%)	107,947.49±4,698.89	1,609.92±45.61	0.056±0.002
	AB4	334 (84.56%)	3 (0.76%)	97,942.40±3,753.44	1,565.42±41.13	0.057±0.002
	M17	333 (84.52%)	10 (2.54%)	108,307.66±4,306.91	1,667.19±47.13	0.045±0.002
Axodendritic	AB1	23 (4.78%)	27 (5.61%)	80,973.53±14,337.30	1,356.75±147.81	0.037±0.004
	AB2	121 (22.92%)	44 (8.33%)	108,858.10±7,942.19	1,563.64±64.53	0.043±0.002
	AB3	22 (5.43%)	32 (7.90%)	107,971.89±17,989.34	1,591.62±172.83	0.038±0.005
	AB4	20 (5.06%)	38 (9.62%)	151,864.86±20,111.74	2,067.97±192.79	0.047±0.006
	M17	16 (4.06%)	35 (8.88%)	117,406.98±20,556.63	1,825.06±192.09	0.031±0.003

Supplementary file 1V.
Ultrastructural data regarding the postsynaptic target in the deep part of the *stratum pyramidale* of CA1 per case. AS: asymmetric synapses; SAS: synaptic apposition surface; sem: standard error of the mean; SS: symmetric synapses.

The following significant differences were observed between cases:

- AB1-AB2 (χ^2, p=1.00x10^{-17})
  - Axospinous AS (χ^2, 4.666x10^{-13})
  - Axodendritic AS (χ^2, p=1.00x10^{-17})
- AB2-AB3 (χ^2, p=4.801x10^{-12})
  - Axospinous AS (χ^2, p=1.301x10^{-10})
  - Axodendritic AS (χ^2, p=2.400x10^{-15})

- AB2-AB4 (χ^2, p=3.187x10^{-12})
  - Axospinous AS (χ^2, p=2.804x10^{-9})
  - Axodendritic AS (χ^2, p=6.000x10^{-16})

- AB2-M17 (χ^2, p=4.500x10^{-15})
  - Axospinous AS (χ^2, p=2.899x10^{-9})
  - Axodendritic AS (χ^2, p=1.000x10^{-17}).

STRATUM PYRAMIDALE (SUP)							
		No. AS	No. SS	SAS Area of AS (nm^2; mean±sem)	SAS Perimeter of AS (nm; mean±sem)	SAS Curvature of AS (mean±sem)
Axospinous	AB1	772 (92.12%)	3 (0.36%)	73,989.28±1,751.81	1,358.47±20.92	0.054±0.001
	AB2	314 (83.96%)	0 (0.00%)	88,278.10±4,154.22	1,426.42±42.29	0.050±0.002
	AB3	389 (84.93%)	0 (0.00%)	95,024.61±3,806.30	1,548.99±39.25	0.053±0.002
	AB4	314 (82.41%)	6 (1.57%)	127,284.99±5,364.09	1,794.53±53.55	0.057±0.002
	M17	395 (89.37%)	8 (1.81%)	123,090.76±5,013.11	1,813.85±52.07	0.052±0.002
Axodendritic	AB1	36 (4.30%)	27 (3.22%)	67,033.19±8,260.31	1,225.02±79.48	0.052±0.005
	AB2	26 (6.95%)	34 (9.09%)	49,507.36±8,780.47	1,062.92±131.20	0.057±0.007
	AB3	41 (8.95%)	28 (6.11%)	95,079.45±12,194.84	1,548.29±112.02	0.049±0.005
	AB4	21 (5.51%)	40 (10.50%)	114,406.11±14,319.52	1,683.78±154.54	0.054±0.009
	M17	7 (1.58%)	32 (7.24%)	90,055.03±31,084.26	1,442.59±225.25	0.031±0.001

Supplementary file 1W.

Ultrastructural data regarding the postsynaptic target in the superficial part of the *stratum pyramidale* of CA1 per case. AS: asymmetric synapses; SAS: synaptic apposition surface; sem: standard error of the mean; SS: symmetric synapses.

The following significant differences were observed between cases:

- AB1-AB2 (χ^2, p=1.939x10^{-5})
    - Axospinous AS (χ^2, p=3.815x10^{-5})
- AB1-AB3 (χ^2, p= 0.0003)
    - Axospinous AS (χ^2, p=0.0001)
- AB1-AB4 (χ^2, p=1.901x10^{-7})
    - Axospinous AS (χ^2, p=1.414x10^{-6})

- AB2-M17 (χ^2, $p=4.564x10^{-5}$)
    - Axodendritic AS (χ^2, $p=0.0001$)
- AB-M17 (χ^2, $p=7.562x10^{-7}$)
    - Axospinous AS (χ^2, $p=6.692x10^{-7}$).

STRATUM RADIATUM						
		No. AS	No. SS	SAS Area of AS (nm^2; mean±sem)	SAS Perimeter of AS (nm; mean±sem)	SAS Curvature of AS (mean±sem)
Axospinous	AB1	406 (87.31%)	4 (0.86%)	78,615.15±2,747.63	1,369.75±28.28	0.052±0.002
	AB2	159 (67.09%)	1 (0.42%)	83,659.03±7,027.88	1,414.65±75.42	0.056±0.003
	AB3	326 (80.49%)	0 (0.00%)	91,889.20±4,501.61	1,513.55±48.31	0.054±0.002
	AB4	188 (72.31%)	0 (0.00%)	119,670.82±6,464.90	1,714.99±67.55	0.058±0.002
	M17	199 (75.38%)	1 (0.38%)	137,465.45±9,118.34	1,917.34±88.21	0.051±0.003
Axodendritic	AB1	34 (7.31%)	21 (4.52%)	83,910.48±13,618.63	1,273.60±117.31	0.046±0.004
	AB2	56 (23.63%)	21 (8.86%)	69,323.01±5,321.07	1,310.21±74.27	0.052±0.003
	AB3	40 (9.88%)	39 (9.63%)	73,100.66±9,204.52	1,302.21±92.32	0.042±0.003
	AB4	35 (13.46%)	37 (14.23%)	81,730.12±12,154.13	1,354.04±129.23	0.057±0.006
	M17	23 (8.71%)	41 (15.53%)	191,416.26±34,376.21	2,333.32±351.32	0.040±0.005

Supplementary file 1X.
Ultrastructural data regarding the postsynaptic target in the *stratum radiatum* of CA1 per case. AS: asymmetric synapses; SAS: synaptic apposition surface; sem: standard error of the mean; SS: symmetric synapses.

The following significant differences were observed between cases:

- AB1-AB2 (χ^2, p=5.799x10^{-10})
    - Axospinous AS (χ^2, p=7.427x10^{-10})
    - Axodendritic AS (χ^2, p=3.899x10^{-8})
- AB1-AB4 (χ^2, p=3.042x10^{-7})
    - Axospinous AS (χ^2, p=1.030x10^{-6})
    - Axodendritic SS (χ^2, p=1.099x10^{-5})

- AB1-M17 (χ^2, p=3.811x10^{-6})
  - Axospinous AS (χ^2, p=5.715x10^{-5})
  - Axodendritic SS (χ^2, p=6.787x10^{-7})
- AB2-AB3 (χ^2, p=2.195x10^{-5})
  - Axospinous AS (χ^2, p=0.0002)
  - Axodendritic AS (χ^2, p=5.712x10^{-6})

- AB2-M17 (χ^2, p=3.466x10^{-5})
  - Axodendritic AS (χ^2, p=4.617x10^{-6}).

STRATUM LACUNOSUM-MOLECULARE						
		No. AS	No. SS	SAS Area of AS (nm^2; mean±sem)	SAS Perimeter of AS (nm; mean±sem)	SAS Curvature of AS (mean±sem)
Axospinous	AB1	257 (76.95%)	6 (1.80%)	83,065.94±3,321.38	1,404.69±40.24	0.045±0.002
	AB2	69 (42.33%)	0 (0.00%)	108,918.10±8,387.64	1,668.96±97.55	0.046±0.004
	AB3	220 (59.30%)	11 (2.96%)	80,266.24±3,551.68	1,437.99±41.37	0.047±0.003
	AB4	132 (50.57%)	9 (3.45%)	109,428.06±6,696.00	1,553.85±65.44	0.042±0.003
	M17	82 (39.23%)	5 (2.39%)	108,727.58±6,012.23	1,770.74±85.69	0.037±0.002
Axodendritic	AB1	40 (11.98%)	31 (9.28%)	171,377.95±19,122.35	2,163.14±182.35	0.049±0.006
	AB2	54 (33.13%)	40 (24.54%)	167,765.83±13,316.03	2,037.10±102.68	0.042±0.005
	AB3	86 (23.18%)	54 (14.56%)	125,214.64±10,453.62	1,786.71±99.75	0.050±0.004
	AB4	69 (26.44%)	51 (19.54%)	183,806.73±14,303.31	2,233.71±125.27	0.046±0.003
	M17	51 (24.40%)	71 (33.97%)	149,766.91±11,574.26	1,987.54±108.43	0.041±0.004

Supplementary file 1Y.
Ultrastructural data regarding the postsynaptic target in the *stratum radiatum* of CA1 per case. AS: asymmetric synapses; SAS: synaptic apposition surface; sem: standard error of the mean; SS: symmetric synapses.

The following significant differences were observed between cases:

- AB1-AB2 (χ^2, p=1.000x10^{-17})
  - Axospinous AS (χ^2, p=7.100x10^{-15})
  - Axodendritic AS (χ^2, p=4.538x10^{-8})
  - Axodendritic SS (χ^2, p=1.393x10^{-6})
- AB1-AB3 (χ^2, p=1.221x10^{-5})
  - Axospinous AS (χ^2, p=5.428x10^{-7})

- Axodendritic AS (χ^2, p=0.0001)

- AB1-AB4 (χ^2, $p=8.884x10^{-10}$)
  - Axospinous AS (χ^2, $p=2.774x10^{-11}$)
  - Axodendritic AS (χ^2, $p=9.745x10^{-6}$)
  - Axodendritic SS (χ^2, $p=9.635x10^{-5}$)
- AB1-M17 (χ^2, $p=1.000x10^{-17}$)
  - Axospinous AS (χ^2, $p=1.000x10^{-17}$)
  - Axodendritic AS (χ^2, p=0.0002)
  - Axodendritic SS (χ^2, $p=2.737x10^{-13}$)
- AB2-AB3 (χ^2, $p=8.108x10^{-5}$)
  - Axospinous AS (χ^2, p=0.0003)
- AB3-M17 (χ^2, $p=2.016x10^{-7}$)
  - Axospinous AS (χ^2, $p=4.169x10^{-6}$)
  - Axodendritic SS (χ^2, $p=1.011x10^{-7}$).

STRATUM ORIENS								
		No. AS	No. SS	% AS	% SS	SAS Area of AS (nm^2; mean±sem)	SAS Perimeter of AS (nm; mean±sem)	SAS Curvature of AS (mean±sem)
Macular	AB1	329	14	82.46%	70.00%	71,579.24±1,967.39	1,193.81±18.81	0.043±0.001
	AB2	864	40	89.91%	83.33%	59,814.16±1,365.19	1,115.07±13.42	0.042±0.001
	AB3	393	33	88.12%	82.50%	85,542.47±3,569.38	1,410.09±38.35	0.044±0.001
	AB4	252	13	72.83%	86.67%	84,867.52±3,346.80	1,407.83±33.93	0.048±0.002
	M17	442	33	89.11%	76.74%	69,102.99±1,881.60	1,288.68±21.82	0.037±0.001
Horseshoe-shaped	AB1	17	6	4.26%	30.00%	183,219.77±16,801.10	3,085.08±246.51	0.082±0.014
	AB2	29	5	3.02%	10.42%	182,127.32±9,673.00	2,746.61±128.61	0.076±0.008
	AB3	14	3	3.14%	7.50%	137,568.89±23,446.87	2,325.37±398.38	0.049±0.006
	AB4	9	1	2.60%	6.67%	167,379.41±26,519.90	2,699.52±397.93	0.060±0.007
	M17	17	7	3.43%	16.28%	185,560.52±20,627.61	3,079.68±303.30	0.059±0.011
Perforated	AB1	50	0	12.53%	0.00%	197,825.31±10,973.68	2,395.02±119.56	0.054±0.003
	AB2	62	1	6.45%	2.08%	208,917.63±9,628.77	2,658.75±120.75	0.055±0.004
	AB3	26	3	5.83%	7.50%	151,181.17±26,095.46	2,212.36±363.18	0.054±0.009
	AB4	80	1	23.12%	6.67%	163,022.68±12,781.49	2,108.82±114.20	0.054±0.003
	M17	32	3	6.45%	6.98%	223,945.46±16,074.55	2,756.46±134.44	0.058±0.005
Fragmented	AB1	3	0	0.75%	0.00%	229,404.54±44,724.53	2,978.62±827.78	0.047±0.003
	AB2	6	2	0.62%	4.17%	187,914.30±42,592.67	1,978.91±285.29	0.131±0.027
	AB3	13	1	2.91%	2.50%	221,662.67±37,955.74	2,365.06±281.85	0.087±0.022
	AB4	5	0	1.45%	0.00%	253,460.37±50,951.13	2,519.40±548.65	0.127±0.049
	M17	5	0	1.01%	0.00%	222,988.12±54,432.34	2,871.76±773.04	0.104±0.027

Supplementary file 1Z.
Ultrastructural data regarding the shape of the synaptic junction in the *stratum oriens* of CA1 per case. AS: asymmetric synapses; SAS: synaptic apposition surface; sem: standard error of the mean; SS: symmetric synapses.

The following significant differences were observed between cases (all non-macular synaptic types were grouped into a single category for contingency analysis):

- AB1-AB2:
  - AS Macular-AS Non-macular (χ^2, p=0.0003)
- AB2-AB4:
  - AS Macular-AS Non-macular (χ^2, p=3.300x10^{-15})
- AB3-AB4
  - AS Macular-AS Non-macular (χ^2, p=6.503x10^{-8})
- AB4-M17
  - AS Macular-AS Non-macular (χ^2, p=1.480x10^{-9}).

STRATUM PYRAMIDALE (DEEP)								
		No. AS	No. SS	% AS	% SS	SAS Area of AS (nm^2; mean±sem)	SAS Perimeter of AS (nm; mean±sem)	SAS Curvature of AS (mean±sem)
Macular	AB1	449	37	85.04%	82.22%	78,730.15±2,027.51	1,283.49±19.06	0.042±0.001
	AB2	1,070	85	91.61%	80.58%	68,976.39±1,583.69	1,209.09±15.29	0.041±0.001
	AB3	598	20	86.17%	51.28%	73,778.67±2,017.49	1,294.03±20.01	0.050±0.001
	AB4	532	38	75.14%	82.61%	68,926.88±1,552.90	1,257.46±16.92	0.050±0.001
	M17	645	37	85.89%	77.08%	76,324.38±1,742.54	1,316.01±17.47	0.039±0.001
Horseshoe-shaped	AB1	31	3	5.87%	6.67%	149,675.87±16,501.34	2,678.37±173.76	0.063±0.007
	AB2	29	13	2.48%	12.62%	174,669.50±13,105.00	2,826.08±170.45	0.075±0.009
	AB3	22	10	3.17%	25.64%	215,452.94±23,446.74	3,247.15±244.84	0.101±0.014
	AB4	27	3	3.81%	6.52%	180,541.81±14,825.42	3,214.16±173.74	0.101±0.013
	M17	32	10	4.26%	20.83%	209,129.34±19,899.44	3,265.64±218.18	0.092±0.012
Perforated	AB1	44	4	8.33%	8.89%	176,386.67±11,306.29	2,244.43±111.14	0.055±0.003
	AB2	58	6	4.97%	5.83%	232,495.35±12,747.71	2,767.16±110.76	0.060±0.004
	AB3	52	8	7.49%	20.51%	223,263.55±12,861.35	2,704.16±141.77	0.070±0.008
	AB4	134	5	18.93%	10.87%	198,734.15±7,530.39	2,546.44±94.12	0.065±0.004
	M17	69	1	9.19%	2.08%	197,780.76±12,455.03	2,541.75±135.32	0.058±0.004
Fragmented	AB1	4	1	0.76%	2.22%	277,288.60±50,954.51	2,101.88±361.68	0.099±0.043
	AB2	11	1	0.94%	0.97%	211,369.76±32,166.87	1,956.33±217.05	0.084±0.016
	AB3	22	1	3.17%	2.56%	315,335.94±22,539.18	2,640.33±149.18	0.130±0.014
	AB4	15	0	2.12%	0.00%	262,814.40±20,115.67	2,632.68±170.81	0.155±0.024
	M17	5	0	0.67%	0.00%	307,082.60±56,381.11	2,512.75±474.32	0.134±0.019

Suplementary file 1AA.
Ultrastructural data regarding the shape of the synaptic junction in the deep part of the *stratum pyramidale* of CA1 per case. AS: asymmetric synapses; SAS: synaptic apposition surface; sem: standard error of the mean; SS: symmetric synapses.

The following significant differences were observed between cases (all non-macular synaptic types were grouped into a single category for contingency analysis):

- AB1-AB2:
  - AS Macular-AS Non-macular (χ^2, p=$7.211x10^{-5}$)
- AB1-AB4:
  - AS Macular-AS Non-macular (χ^2, p=$1.920x10^{-5}$)
- AB2-AB3:
  - AS Macular-AS Non-macular (χ^2, p=0.0003)
- AB2-AB4:
  - AS Macular-AS Non-macular (χ^2, p=$1.000x10^{-17}$)
- AB2-M17:
  - AS Macular-AS Non-macular (χ^2, p=0.0001)
- AB3-AB4:
  - AS Macular-AS Non-macular (χ^2, p=$1.756x10^{-7}$)
- AB4-M17:
  - AS Macular-AS Non-macular (χ^2, p=$2.077x10^{-7}$).

STRATUM PYRAMIDALE (SUP)								
		No. AS	No. SS	% AS	% SS	SAS Area of AS (nm^2; mean±sem)	SAS Perimeter of AS (nm; mean±sem)	SAS Curvature of AS (mean±sem)
Macular	AB1	871	29	81.94%	93.55%	57,496.39±1,054.48	1,153.86±11.86	0.047±0.001
	AB2	1,149	34	92.51%	79.07%	65,443.73±1,391.25	1,192.36±13.57	0.051±0.001
	AB3	1,061	24	89.92%	80.00%	65,334.44±1,252.92	1,247.35±13.50	0.047±0.001
	AB4	542	38	77.76%	77.55%	84,802.11±1,985.86	1,381.46±18.63	0.052±0.001
	M17	894	39	89.31%	90.70%	81,656.02±1,662.13	1,406.95±17.33	0.044±0.001
Horseshoe-shaped	AB1	73	1	6.87%	3.23%	119,267.30±7,495.94	2,255.35±99.02	0.074±0.005
	AB2	37	4	2.98%	9.30%	254,061.00±15,689.07	3,359.05±143.48	0.096±0.006
	AB3	47	4	3.98%	13.33%	205,616.90±10,852.09	3,172.30±147.89	0.101±0.009
	AB4	25	4	3.59%	8.16%	245,836.20±24,434.06	3,676.85±294.94	0.075±0.009
	M17	30	3	3.00%	6.98%	241,140.40±22,881.63	3,684.42±308.37	0.115±0.014
Perforated	AB1	105	1	9.88%	3.23%	130,096.60±5,612.62	1,995.67±62.80	0.081±0.005
	AB2	47	1	3.78%	2.33%	235,325.60±10,943.00	2,797.07±117.30	0.084±0.009
	AB3	43	0	3.64%	0.00%	233,736.20±13,618.83	2,738.23±126.19	0.084±0.008
	AB4	112	3	16.07%	6.12%	233,271.20±8,764.45	2,805.01±91.58	0.067±0.003
	M17	64	1	6.39%	2.33%	292,952.60±16,089.11	3,463.83±186.25	0.075±0.006
Fragmented	AB1	14	0	1.32%	0.00%	212,851.50±26,291.48	2,314.15±253.01	0.138±0.026
	AB2	9	4	0.72%	9.30%	203,875.90±33,946.89	2,238.76±256.80	0.072±0.011
	AB3	29	2	2.46%	6.67%	244,916.90±17,971.50	2,370.95±138.85	0.117±0.012
	AB4	18	4	2.58%	8.16%	324,793.90±21,657.34	2,725.81±189.21	0.157±0.013
	M17	13	0	1.30%	0.00%	347,186.20±25,769.52	3,167.86±262.15	0.171±0.036

Supplementary file 1AB.
Ultrastructural data regarding the shape of the synaptic junction in the superficial part of the *stratum pyramidale* of CA1 per case. AS: asymmetric synapses; SAS: synaptic apposition surface; sem: standard error of the mean; SS: symmetric synapses.

The following significant differences were observed between cases (all non-macular synaptic types were grouped into a single category for contingency analysis):

- AB1-AB2:
  - AS Macular-AS Non-macular (χ^2, p=1.900x10^{-15})
- AB1-AB3:
  - AS Macular-AS Non-macular (χ^2, p=1.613x10^{-8})
- AB1-M17:
  - AS Macular-AS Non-macular (χ^2, p=1.843x10^{-6})
- AB2-AB4:
  - AS Macular-AS Non-macular (χ^2, p=1.000x10^{-17})
- AB3-AB4:
  - AS Macular-AS Non-macular (χ^2, p=1.767x10^{-13})
- AB4-M17:
  - AS Macular-AS Non-macular (χ^2, p=1.496x10^{-11}).

STRATUM RADIATUM								
		No. AS	No. SS	% AS	% SS	SAS Area of AS (nm^2; mean±sem)	SAS Perimeter of AS (nm; mean±sem)	SAS Curvature of AS (mean±sem)
Macular	AB1	568	23	87.95%	92.00%	68,441.72±1,880.37	1,252.81±19.74	0.050±0.001
	AB2	809	21	90.12%	87.50%	57,905.52±1,741.87	1,126.16±17.11	0.053±0.001
	AB3	946	36	90.34%	92.31%	59,199.20±1,234.64	1,159.89±13.48	0.045±0.001
	AB4	436	34	76.55%	64.29%	72,845.49±2,015.31	1,241.70±18.57	0.056±0.002
	M17	579	27	84.87%	80.95%	70,869.88±1,911.78	1,245.05±18.57	0.036±0.001
Horseshoe-shaped	AB1	34	0	5.06%	0.00%	122,773.40±12,538.54	2,018.18±144.47	0.062±0.010
	AB2	39	1	4.34%	4.17%	198,204.30±12,709.33	3,219.84±214.07	0.070±0.005
	AB3	43	1	4.05%	2.56%	183,521.60±12,386.53	2,706.35±126.51	0.069±0.006
	AB4	20	5	4.07%	11.90%	173,804.50±20,244.18	3,084.70±303.97	0.074±0.008
	M17	14	14	3.92%	33.33%	227,324.70±23,151.54	3,243.59±206.94	0.078±0.011
Perforated	AB1	36	1	5.51%	4.00%	127,297.40±12,183.28	1,828.05±112.44	0.066±0.009
	AB2	40	0	4.34%	0.00%	212,574.60±13,737.09	2,659.22±148.73	0.086±0.001
	AB3	31	0	2.85%	0.00%	194,467.80±11,623.65	2,808.49±193.32	0.083±0.013
	AB4	99	1	16.29%	2.38%	197,019.10±8,629.29	2,524.29±96.59	0.071±0.004
	M17	68	1	9.66%	2.38%	277,764.10±16,430.86	3,370.50±180.99	0.074±0.006
Fragmented	AB1	9	1	1.49%	4.00%	203,063.30±48,386.31	2,197.56±345.52	0.098±0.044
	AB2	9	2	1.19%	8.33%	220,118.50±24,204.66	2,163.58±163.91	0.105±0.018
	AB3	28	2	2.76%	5.13%	213,947.90±20,066.83	2,211.08±178.18	0.088±0.012
	AB4	17	2	3.09%	4.76%	332,424.10±26,011.06	3,113.61±261.65	0.162±0.022
	M17	11	0	1.54%	0.00%	377,822.90±28,842.89	3,730.26±284.32	0.139±0.018

Supplementary file 1AC.
Ultrastructural data attending to the shape of the synaptic junction in the *stratum radiatum* of CA1 per case. AS: asymmetric synapses; SAS: synaptic apposition surface; sem: standard error of the mean; SS: symmetric synapses.

The following significant differences were observed between cases (all non-macular synaptic types were grouped into a single category for contingency analysis):

- AB-AB4:
  - AS Macular-AS Non-macular (χ^2, p=1.673x10^{-7})
- AB2-AB4:
  - AS Macular-AS Non-macular (χ^2, p=1.242x10^{-13})
- AB3-AB4:
  - AS Macular-AS Non-macular (χ^2, p=2.300x10-^{15})
- AB4-M17:
  - AS Macular-AS Non-macular (χ^2, p=7.151x10^{-6}).

STRATUM LACUNOSUM-MOLECULARE								
		No. AS	No. SS	% AS	% SS	SAS Area of AS (nm^2; mean±sem)	SAS Perimeter of AS (nm; mean±sem)	SAS Curvature of AS (mean±sem)
Macular	AB1	372	41	81.76%	95.35%	68,113.92±2,188.68	1,206.36±20.98	0.045±0.002
	AB2	453	24	84.99%	60.00%	75,667.98±2,563.19	1,273.63±21.51	0.040±0.001
	AB3	600	52	85.47%	74.29%	63,852.28±1,708.22	1,212.36±16.96	0.045±0.001
	AB4	313	65	72.96%	92.86%	78,330.93±2,507.34	1,254.88±24.03	0.038±0.002
	M17	422	76	83.90%	81.72%	74,589.08±2,372.56	1,289.81±23.98	0.036±0.001
Horseshoe-shaped	AB1	45	1	9.89%	2.33%	162,197.29±11,223.32	2,649.59±120.03	0.053±0.007
	AB2	28	12	5.25%	30.00%	201,575.63±13,044.70	2,964.05±157.15	0.054±0.005
	AB3	43	9	6.13%	12.86%	129,287.65±7,785.24	2,422.88±108.91	0.046±0.003
	AB4	40	4	9.32%	5.71%	164,325.03±11,131.14	2,636.94±128.61	0.062±0.006
	M17	33	11	6.56%	11.83%	144,353.95±8,840.51	2,691.56±144.27	0.055±0.006
Perforated	AB1	35	1	7.69%	2.33%	198,469.66±13,753.14	2,441.51±133.31	0.049±0.004
	AB2	47	3	8.82%	7.50%	245,905.08±13,748.10	2,805.81±111.97	0.057±0.006
	AB3	42	5	5.98%	7.14%	164,625.20±12,911.39	2,339.99±148.91	0.060±0.006
	AB4	62	1	14.45%	1.43%	225,485.79±12,755.30	2,612.39±115.85	0.059±0.005
	M17	36	3	7.16%	3.23%	171,401.99±10,472.22	2,515.02±143.45	0.060±0.007
Fragmented	AB1	3	0	0.66%	0.00%	198,066.31±42,515.28	2,050.85±367.29	0.114±0.060
	AB2	5	1	0.94%	2.50%	224,196.15±11,217.44	2,356.51±210.70	0.067±0.017
	AB3	17	4	2.42%	5.71%	177,905.27±18,503.35	2,100.79±151.01	0.067±0.008
	AB4	14	0	3.26%	0.00%	218,888.89±28,799.14	2,026.17±236.32	0.072±0.007
	M17	12	3	2.39%	3.23%	195,513.30±19,527.34	2,460.70±209.08	0.063±0.013

Supplementary file 1AD.

Ultrastructural data regarding the shape of the synaptic junction in the *stratum lacunosum-moleculare* of CA1 per case. AS: asymmetric synapses; SAS: synaptic apposition surface; sem: standard error of the mean; SS: symmetric synapses.

The following significant differences were observed between cases (all non-macular synaptic types were grouped into a single category for contingency analysis):

- AB1-AB2:
  - SS Macular-AS Non-macular (χ^2, p=0.0001)
- AB1-AB4:
  - AS Macular-AS Non-macular (χ^2, p=0.0002)
- AB2-AB4:
  - AS Macular-AS Non-macular (χ^2, p=5.796x10^{-6})
  - SS Macular-AS Non-macular (χ^2, p=6.553x10^{-5})
- AB3-AB4:
  - AS Macular-AS Non-macular (χ^2, p=3.761x10^{-7})
- AB4-M17:
  - AS Macular-AS Non-macular (χ^2, p=5.416x10^{-5}).

Case	Sex	Age (years)	Cause of death	Postmortem delay (h)	Neuropathological diagnosis
AB1	Male	45	Lung cancer	<1	No neurological alterations
AB2	Female	53	Pulmonary shock	4	No neurological alterations
AB3	Male	53	Bladder carcinoma	3.5	No neurological alterations
AB4	Female	65	Bone sarcoma	4.5	No neurological alterations
M17	Male	36	Bronchopneumonia	2.5	No neurological alterations

Supplementary file 1AE.

Clinical information regarding the human cases. None of the five subjects had recorded neurological or psychiatric alterations.
