# Supplementary material for: Three-dimensional synaptic organization of the human hippocampal CA1 field
Source: eLife. 2020 Jul 21;9:e57013. doi: 10.7554/eLife.57013 (PMC7375818; doi:10.7554/eLife.57013)
Supplement: Transparent reporting form [file elife-57013-transrepform.pdf]

1st Floor
24 Hills Road
Cambridge CB2 1JP, UK

P 01223 855340
W elifesciences.org
T @elife

eLife's transparent reporting form

We encourage authors to provide detailed information *within their submission* to facilitate the interpretation and replication of experiments. Authors can upload supporting documentation to indicate the use of appropriate reporting guidelines for health-related research (see EQUATOR Network), life science research (see the BioSharing Information Resource), or the ARRIVE guidelines for reporting work involving animal research. Where applicable, authors should refer to any relevant reporting standards documents in this form.

If you have any questions, please consult our Journal Policies and/or contact us: editorial@elifesciences.org.

Sample-size estimation

- You should state whether an appropriate sample size was computed when the study was being designed
- You should state the statistical method of sample size computation and any required assumptions
- If no explicit power analysis was used, you should describe how you decided what sample (replicate) size (number) to use

Please outline where this information can be found within the submission (e.g., sections or figure legends), or explain why this information doesn't apply to your submission:

The inclusion criteria of the human cases used can be found in the Materials and Methods sections. We have used samples coming from five postmortem human brain tissue cases that were selected because the tissue was well-preserved and because this provided the sample size necessary to conduct proper statistical analysis, considering the difficulties involved in obtaining this kind of human tissue.
We have analyzed 75 FIB/SEM stacks of images (3 stacks per layer and case) with a total volume of 29,322 μm^3 and a total number of 19,269 synapses (18,138 asymmetric and 1,131 symmetric) after discarding incomplete synapses. The number of synapses (n) in each analyzed category is indicated in the manuscript in each subsection of the "Results" section, as well as in most figures and in the main and supplementary tables.

Replicates

- You should report how often each experiment was performed
- You should include a definition of biological versus technical replication
- The data obtained should be provided and sufficient information should be provided to indicate the number of independent biological and/or technical replicates
- If you encountered any outliers, you should describe how these were handled
- Criteria for exclusion/inclusion of data should be clearly stated
- High-throughput sequence data should be uploaded before submission, with a private link for reviewers provided (these are available from both GEO and ArrayExpress)

Please outline where this information can be found within the submission (e.g., sections or figure legends), or explain why this information doesn't apply to your submission:

This data is available in the Materials and Methods section. We used five different human brain samples and we acquired three stacks of images per region and subject. Our findings were obtained by using volume electron microscopy methodologies and statistical analyses.

Statistical reporting

- Statistical analysis methods should be described and justified
- Raw data should be presented in figures whenever informative to do so (typically when N per group is less than 10)
- For each experiment, you should identify the statistical tests used, exact values of N, definitions of center, methods of multiple test correction, and dispersion and precision measures (e.g., mean, median, SD, SEM, confidence intervals; and, for the major substantive results, a measure of effect size (e.g., Pearson's r, Cohen's d)
- Report exact p-values wherever possible alongside the summary statistics and 95% confidence intervals. These should be reported for all key questions and not only when the p-value is less than 0.05.

Please outline where this information can be found within the submission (e.g., sections or figure legends), or explain why this information doesn't apply to your submission:

This data is available in the Statistical Analysis subsection in Materials and Methods. This information is also available in each figure legend and table legend when pertinent. Additionally, all data for each case and its statistical analysis is available in the supplementary tables.

(For large datasets, or papers with a very large number of statistical tests, you may upload a single table file with tests, Ns, etc., with reference to sections in the manuscript.)

Group allocation

- Indicate how samples were allocated into experimental groups (in the case of clinical studies, please specify allocation to treatment method); if randomization was used, please also state if restricted randomization was applied
- Indicate if masking was used during group allocation, data collection and/or data analysis

Please outline where this information can be found within the submission (e.g., sections or figure legends), or explain why this information doesn't apply to your submission:

Group allocation was not relevant to our study.

Additional data files ("source data")

- We encourage you to upload relevant additional data files, such as numerical data that are represented as a graph in a figure, or as a summary table
- Where provided, these should be in the most useful format, and they can be uploaded as "Source data" files linked to a main figure or table
- Include model definition files including the full list of parameters used
- Include code used for data analysis (e.g., R, MatLab)
- Avoid stating that data files are "available upon request"

Please indicate the figures or tables for which source data files have been provided:

eLIFE
1st Floor
24 Hills Road
Cambridge CB2 1JP, UK
P 01223 855340
W elifesciences.org
T @elife

All data (per region and case) is available in the main table and supplementary tables of the manuscript, with the exception of raw data per stack of FIB/SEM images, which has been published on the EBRAINS Knowledge Graph (DOI: https://doi.org/10.25493/NRFB-7N5).
